# Investigating Helium Bubble Nucleation and Growth through Simultaneous In-Situ Cryogenic, Ion Implantation, and Environmental Transmission Electron Microscopy

**DOI:** 10.3390/ma12162618

**Published:** 2019-08-16

**Authors:** Caitlin A. Taylor, Samuel Briggs, Graeme Greaves, Anthony Monterrosa, Emily Aradi, Joshua D. Sugar, David B. Robinson, Khalid Hattar, Jonathan A. Hinks

**Affiliations:** 1 Sandia National Laboratories, Albuquerque, NM 87185, USA; 2 Nuclear Science and Engineering, Oregon State University, Corvallis, OR 97331, USA; 3 School of Computing and Engineering, University of Huddersfield, Huddersfield HD1 3DH, UK; 4 Sandia National Laboratories, Livermore, CA 94551, USA

**Keywords:** in-situ, helium implantation, environmental transmission electron microscopy, palladium tritide

## Abstract

Palladium can readily dissociate molecular hydrogen at its surface, and rapidly accept it onto the octahedral sites of its face-centered cubic crystal structure. This can include radioactive tritium. As tritium β-decays with a half-life of 12.3 years, He-3 is generated in the metal lattice, causing significant degradation of the material. Helium bubble evolution at high concentrations can result in blister formation or exfoliation and must therefore be well understood to predict the longevity of materials that absorb tritium. A hydrogen over-pressure must be applied to palladium hydride to prevent hydrogen from desorbing from the metal, making it difficult to study tritium in palladium by methods that involve vacuum, such as electron microscopy. Recent improvements in in-situ ion implantation Transmission Electron Microscopy (TEM) allow for the direct observation of He bubble nucleation and growth in materials. In this work, we present results from preliminary experiments using the new ion implantation Environmental TEM (ETEM) at the University of Huddersfield to observe He bubble nucleation and growth, in-situ, in palladium at cryogenic temperatures in a hydrogen environment. After the initial nucleation phase, bubble diameter remained constant throughout the implantation, but bubble density increased with implantation time. β-phase palladium hydride was not observed to form during the experiments, likely indicating that the cryogenic implantation temperature played a dominating role in the bubble nucleation and growth behavior.

## 1. Introduction

Helium is insoluble in almost all solids and precipitates into nanometer-sized bubbles that can result in mechanical property degradation and eventually fracture. At very high He concentrations (tens of atomic percent), micrometer scale blisters can form, resulting in exfoliation, gas release, or both [1,2]. Bubble nucleation and growth are sensitive to most environmental conditions, including material composition, crystal structure, and temperature.

Palladium-based materials are under consideration for many applications [3], including H_2_ purification, storage, and detection, as well as fuel cell catalysis, due to its ability to easily dissociate molecular H_2_ on its surfaces and incorporate H atoms into octahedral sites in its face-centered cubic (fcc) crystal structure as a metal hydride [4,5,6]. One of these applications is solid-state tritium storage, where ^3^H will decay to ^3^He with a half-life of 12.3 years, causing rapid accumulation of ^3^He in the Pd lattice. Studying ^3^He evolution in PdT_x_ is difficult due to the safety constraints of radiological work, though some microscopy has been performed investigating early stage ^3^He bubble formation (less than one year of aging) [7,8,9]. Over these short aging times, ^3^He bubbles reached 1–2 nm in diameter. While He ion implantation has been utilized as an accelerated aging method to study blister formation in Pd metal at high doses [1,2], He implantation into Pd hydride is difficult in most facilities because a constant H_2_ over-pressure is required to maintain the hydride structure. If the over-pressure is removed, most H will diffuse out of the material [4,5,6].

New capabilities in in-situ ion irradiation allow for direct observation of He bubble nucleation and growth as a function of He implantation dose and temperature [10]. In the new Microscope and Ion Accelerators for Materials Investigations (MIAMI-2) facility at the University of Huddersfield [11], in-situ He implantation has been combined with Environmental Transmission Electron Microscopy (ETEM), which allows for imaging in the presence of milliTorr H_2_ pressures. We have used this combination of capabilities to investigate whether the presence of H_2_ affects the nucleation and early growth of bubbles. We performed much of the work at sub-ambient sample temperatures to increase the H solubility in the sample, but did not observe formation of the concentrated, β-phase Pd hydride, which is difficult to characterize with electron diffraction techniques. α-phase Pd hydride is expected to have formed to some degree under the experimental conditions, but cannot be properly identified using electron diffraction due to its characteristic minute change in lattice parameter Thus, cryogenic temperature likely dominated the observed He bubble nucleation and growth kinetics.

## 2. Materials and Methods

### Specimens and Irradiation Treatment

Palladium wire was purchased from Alfa Aesar (Alfa Aesar, Haverhill, MA, USA) and was annealed prior to TEM sample preparation to cause pre-existing voids identified near the surface to coalesce into larger voids that could not be confused with He bubbles. The wire was annealed at 700 °C for 1.5 h in an evacuated quartz ampoule with a base pressure of 1 × 10^−7^ Torr at the time of sealing. TEM sample preparation was done using the Focused Ion Beam (FIB) method with a FEI Helios Nanolab 660 (ThermoFisher Scientific, Hillsboro, OR, USA). The resulting lamellae were mounted on Mo FIB grids and thinned to electron transparency, with final cleaning steps utilizing a 5 kV accelerating voltage. Samples were then transported and imaged in the Hitachi H-9500 ETEM (Hitachi High-Technologies, Tokyo, Japan) at the MIAMI-2 facility (University of Huddersfield, West Yorkshire, UK) [11]. Unless otherwise stated, all TEM imaging was conducted in a Bright Field (BF) imaging condition with an accelerating voltage of 300 kV. Initial TEM imaging showed a high density of defects, likely resulting from either the FIB procedure or the original wire extrusion process. Since pre-existing defects will affect ^4^He bubble nucleation and hydride formation, the specimens were annealed at 400 °C for one hour in vacuum using a Gatan Model 652 double-tilt heating holder (Gatan, Pleasanton, CA, USA) in an attempt to reduce defect density.

**Figure 1:**
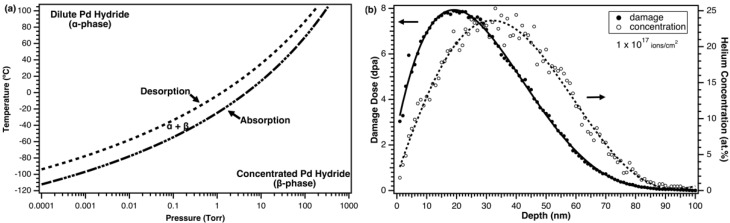
Experimental parameters, including: (**a**) thermodynamic calculations showing when H_2_ is expected to absorb and desorb from pure Pd as a function of temperature and pressure, and (**b**) SRIM prediction, shown for a fluence of 10^17^ ions/cm^2^, of implantation depth, damage dose, and ^4^He concentration for 10 keV ^4^He into Pd at 18.7°. Lines are meant to guide the eye in (**b**).

Thermodynamic calculations [12], shown in Figure 1a, were used to estimate the temperature required to hydride Pd at ETEM relevant pressures (on the order of 10^−2^ Torr). The α-phase has been characterized by a slight unit cell expansion from the fcc Pd lattice of 3.88 Å to 3.89 Å when H/Pd = 0.03. As H_2_ content increases, a new set of fcc lattice reflections form, corresponding to the β-phase, which has a cell constant of 4.02 Å when the α→β transformation is complete (H/Pd~0.57) [13]. This corresponds to a 10% volume expansion, or a 3.6% lattice parameter expansion, compared to Pd metal.

We expect concentrated hydride, or β-phase, to form below the “absorption” line in Figure 1a, and the dilute, or α-phase, to form above the “desorption” line at a given pressure as the temperature is increased. The crystal structure is expected to remain fcc in all cases. The region between the “desorption” and “absorption” lines in Figure 1a consists of α + β-phases [5,6]. These calculations do not include kinetic aspects of hydride formation, which are not well documented for Pd at cryogenic temperatures and may influence the achievement of the hydride phase and final stoichiometry. Furthermore, the thermodynamic data are extrapolated, and are potentially sample-dependent, so we consider Figure 1a to be only an approximate guide.

Samples were cooled to −100 °C in the ETEM using a Gatan Model 636 (Gatan, Pleasanton, CA, USA) liquid nitrogen-cooled cryogenic holder. Thermodynamic calculations (Figure 1a) show that the pressure must be above 6.4 × 10^−4^ Torr to form concentrated β-phase at −100 °C. However, experimental isotherms show that the concentrated β-phase forms above ~1 × 10^−4^ Torr at −196 °C [13]. An H_2_ atmosphere was introduced locally to the specimen. Local specimen pressure was maintained at between 7.5 × 10^−3^ and 2.3 × 10^−2^ Torr while BF imaging video data were collected. Specimen pressure was actively throttled to prevent electron gun pressure from rising to the trip point for the gun valve to close (3.8 × 10^−4^ Torr). Initial experiments were conducted without an ion beam to observe potential microstructural changes associated with the formation of Pd hydride. Palladium hydride formation is characterized by a unit cell expansion [13], so electron diffraction patterns were recorded and utilized to measure the lattice strain in an H_2_ environment at different temperatures. Gas pressure was maintained for 30 min at −100 °C before temperature was increased to −60 °C. Temperature was held there for approximately 20 min before being increased to −20 °C and held for an additional approximately 10 min. Temperature was then reduced back to −100 °C to maximize H_2_ solubility and held for 20 min before beginning He implantation.

Helium implantation was performed using a gas-fed Colutron G-2 ion source (Colutron, Boulder, CO, USA) operating at an accelerating voltage of 10 kV. The ^4^He ion beam had been aligned prior to Pd sample loading using a custom-built Faraday stage. Ion beam intensity was measured to be approximately 4.0 × 10^13^ ions/cm^2^/s at the beginning of irradiation. The Pd sample was irradiated in the H_2_ environment for 42 min to a final nominal ^4^He fluence of 10^17^ ions/cm^2^, all with concurrent collection of BF video data. Ion beam current was monitored throughout via a skimming cup and was observed to drop by only ~3% between the beginning and end of specimen irradiation. Once this final fluence was accomplished, BF images and diffraction patterns were collected from various locations on the specimen. The Monte-Carlo based SRIM code [14] was used to simulate material damage and ion implantation for the given irradiation conditions (Figure 1b). SRIM calculations were performed using an incident ^4^He ion beam at an incoming angle of 18.7°, impinging on Pd metal following the procedure given by Stoller et al. [15]. Displacements per atom (dpa) was calculated using Quick Calculation mode and the phonon.txt output file. A threshold displacement energy of 34 eV [16] and a density of 11.9 g/cm^3^ were used.

Bubble sizes and density were determined, where possible, using ImageJ [17] analysis. Image resolution variations can affect the bubble density analysis by up to an order of magnitude. To maintain consistency, only images in the under-focus condition were used. Bubbles were confirmed using both under- and over-focus images. A sample set of under- and over-focus images is provided in a Supplemental file. Images were all converted to a 1712 × 1712 resolution (used for in-situ video) before analysis and the same procedure was utilized on all images. Image analysis procedure was as follows: (1) Gaussian Blur with radius of 3, (2) Normalize Local Contrast with radii of 20 pixels, (3) invert to make bubbles appear dark, (4) “Mexican Hat” Filter with radius of 4 or 5, (5) threshold the entire image, and finally (6) Analyze Particles of area 0–infinity and circularity set to 0.6–1. Data were exported and bubbles with less than 1 nm diameter, the approximate TEM resolution limit, were removed from the dataset. Only average bubble size is provided because the standard deviation is small, usually less than 0.2 nm.

## 3. Results

### 3.1. Exposure to H_2_ at Cryogenic Temperature

**Figure 2:**
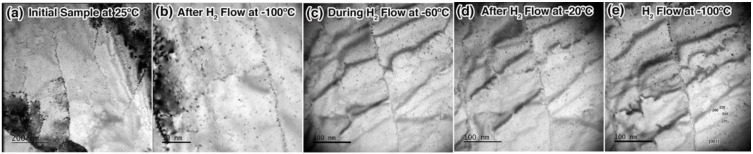
In-situ TEM images of the Pd sample during pre-implantation H_2_ exposure. Images are in sequential order and show the sample (**a**) initially, (**b**) after 25 min of H_2_ exposure at −100 °C, (**c**) during the 14 min of H_2_ exposure at −60 °C, (**d**) after 7 min of H_2_ flow at −20 °C, and (**e**) after cooling back to −100 °C in H_2_ for ^4^He implantation.

To determine the effects of an H_2_ atmosphere on the Pd sample at cryogenic temperatures, a sample was subjected to an H_2_ environment at temperatures between −100 °C and −20 °C. As shown in Figure 2, no significant microstructural changes were observed due to H_2_ alone. Selected Area Electron Diffraction (SAED) patterns were taken after each step. Experimental error in the SAED was not explicitly quantified, but is expected to be large in these experiments due to slight variation in tilt angle and sample height with variation in H_2_ flow rate or temperature. The degree of hydride phase formation, which is characterized by a 3.6% lattice expansion in PdH_0.57_, was therefore unquantifiable in these experiments. Image contrast changes apparent in Figure 2 are due to the sample bending during the temperature cycles.

### 3.2. In-situ Helium Implantation and Annealing in H_2_

**Figure 3:**
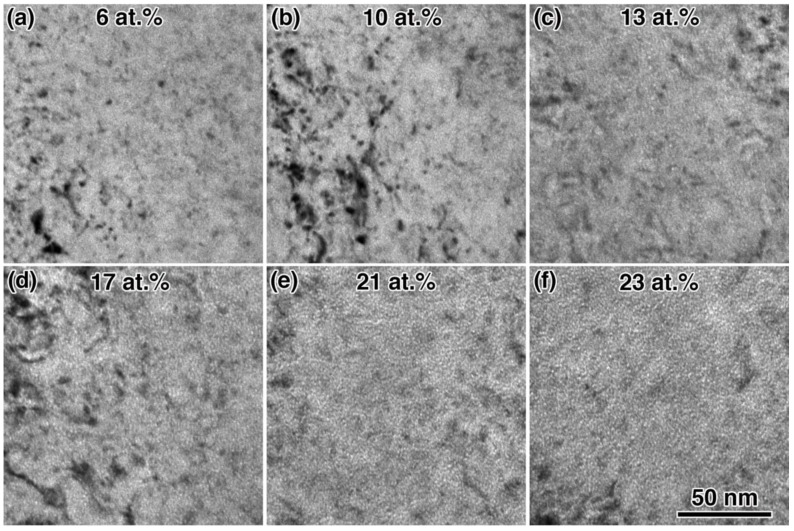
BF in-situ TEM images showing He bubble evolution during implantation at −100 °C under H_2_ gas flow from peak concentrations of (**a**) 6 to (**f**) 23 at.%. The images were taken in Fresnel under-focus imaging condition, so bubbles appear as small white circles.

**Figure 4:**
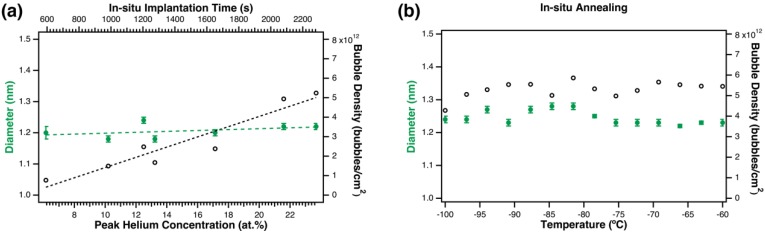
Bubble size and density changes (**a**) during in-situ implantation at −100 °C in H_2_ gas, and (**b**) during annealing from −100 °C to −60 °C. Data points in (**a**) were measured from the images in Figure 3. Bubble diameter is shown as solid green circles, and bubble density is shown as empty black circles. Linear fits are shown for the data in (**a**).

Helium bubble evolution was observed and characterized in-situ during implantation in an H_2_ environment at −100 °C. Figure 3a shows initial observation of ^4^He bubbles after implantation to a peak concentration of 6 at.%. All reported concentration values assume no escape of He from the TEM foil. Bubbles initially had low areal-density and were approximately 1.2 nm in diameter, similar to the microstructure that was observed in the preliminary experiments at room temperature. Larger bubbles were observed in some areas, but bubble nucleation was generally homogenously distributed and uniform in size throughout the implantation. Bubble density visibly increased with increasing implantation dose as shown in Figure 3b–f. Figure 4a shows the measured bubble diameter and density changes as a function of He concentration. Bubble size remained constant during the implantation, but areal bubble density was observed to increase with implantation time, starting at 8 × 10^11^ bubbles/cm^2^ in Figure 3a at 6 at.%, and reaching a density of 5 × 10^12^ bubbles/cm^2^ in Figure 3f at 23 at.%. Bubble density change as a function of implantation dose was determined using a linear fit, where the slope = 2.7 × 10^11^ bubbles/cm^2^/at.% and the intercept = −1.2 × 10^12^ bubbles/cm^2^.

After implantation, the sample was heated in-situ from −100 °C to 0 °C in H_2_ gas. According to Figure 1a, most H_2_ should be desorbed from the sample during this heating process. Bubble size and density were characterized during the annealing, when possible, and are summarized in Figure 4b. The sample was annealed to −60 °C in 390 s (0.10 °C/s), held at −60 °C for approximately 10 min, then ramped from −60 °C to 0 °C in approximately 3 min. Images recorded from −60 °C to 0 °C had too much dynamic contrast evolution for bubble size analysis, so two post-annealing images were analyzed and the results averaged to obtain a final density of 7 × 10^12^ bubbles/cm^2^ and a final diameter of 1.3 nm. No significant changes in bubble diameter were observed during annealing up to 0 °C. No significant changes in bubble density were observed under annealing from −100 °C to −60 °C, but the bubble density did appear to increase from 6 × 10^12^ bubbles/cm^2^ to 7 × 10^12^ bubbles/cm^2^ after annealing from −60 °C to 0 °C (not shown in figure). Uncertainty is not provided for bubble density measurements because a single density value was calculated from each image. 

## 4. Discussion

Helium bubble diameter was observed to remain constant during the cryogenic in-situ implantation in the presence of H_2_, but bubble density increased as a function of time. Even though β-phase hydride formation could not be identified within experimental error, the lower concentration α-phase hydride likely formed and could impact bubble evolution. Although H_2_ may influence the nucleation and growth of He bubbles in Pd, the H concentration is very low in α-phase (H/Pd = 0.03), so cryogenic implantation temperature is thought to be a more-likely dominating factor. Very little literature exists on He implantation in a H_2_ environment or on cryogenic He implantation, so this section will discuss, in relation to this work, (1) a comparison of this work with previous studies on He bubble evolution in Pd, (2) theory on He bubble growth at cryogenic temperature, and (3) how H-He interactions may affect the results.

### 4.1. Comparison with Data on He bubble Nucleation in Pd and PdT_0.6_

Although very little microscopy work has been done on fcc metals implanted with He at cryogenic temperatures, He bubble formation has been studied in aged Pd tritide, PdT_0.6_. Tritium decay does not induce displacement damage in the lattice, so the vacancy concentration is limited to thermal vacancies, although H can stabilize vacancies, and formation of β-phase hydride can cause microstructural changes [18]. Bubbles are at equilibrium when the He pressure inside the bubble equals the surface tension of the host material, *p* = 2*γ*/*r*, where *p* is the He pressure, *γ* is the surface energy of the host material, and *r* is the bubble radius [19]. Bubbles formed at room temperature in PdT_0.6_ are typically highly over-pressurized. He bubbles reach 1–2 nm in diameter in the first 8 months (1.34 at.% He) of storage. Bubble density estimates vary widely due to film thickness measurement error, overlapping bubbles, and the possible presence of bubble sizes near or below the resolution limit of the microscope, but values of 10^17^–10^19^ bubbles/cm^3^ are reported for aging times of 2–8 months [7,8,9]. If a thickness of 50 nm is assumed for the samples implanted in this work, the density varied from 10^17^–10^18^ bubbles/cm^3^ with implantation dose from 2 × 10^16^ to 9 × 10^16^ ions/cm^2^. Bubble diameter did not vary with implantation dose in this work and is similar to that observed in aged PdT_0.6_. Nuclear Magnetic Resonance (NMR) has been used to measure the phase and pressure of He bubbles in aged PdT_0.6_. After annealing samples aged for 1 year, solid He diffusion was observed below −73 °C, and melting was observed from −73 °C to 7 °C, with corresponding bubble pressures of 6–11 GPa, and an average density of 120 He/nm^3^ within a bubble [20]. Aging for 8 years, followed by multiple deuterium exchanges to remove the tritium, resulted in an average density of 90 He/nm^3^ and the presumed coexistence of liquid and solid phases from −230 °C to −133 °C [21].

### 4.2. Comparison with Theory of He Bubble Nucleation and Growth at Cryogenic Temperatures

Several mechanisms could result in a lack of bubble growth with implantation time. Low He diffusivity at cryogenic temperature will contribute to a lower nucleation and growth rate. Low diffusivity may promote a higher bubble nucleation rate, but a lower bubble growth rate due to He becoming trapped very near its implantation site. Bubble diameter remained constant at 1.2 nm after the initial observation of He bubbles at a fluence of 2.35 × 10^16^ ions/cm^2^ (6 at.%), but bubble density increased with implantation dose. Since the He implantation profile, shown in Figure 1b, results in lower concentrations near the surfaces, nucleation will take longer in these regions than at the center of the foil where the He concentration is highest. TEM imaging captures all the material within the thickness of the sample, which could result in a visible increase in bubble density as nucleation occurs first in the higher concentration, and subsequently in the lower concentration regions. This may contribute to an experimentally measured increase in areal bubble density.

At cryogenic temperatures, the low thermal vacancy concentration prohibits cavity growth by absorption of thermal vacancies, which cause rapid cavity growth at elevated temperatures. If the mobility of irradiation- or hydrogen-induced vacancies is low, bubble growth can only occur from dislocation loop punching. Assuming a Pd vacancy migration value of 0.63 eV [22], Pd vacancies are not mobile over the timescale of these experiments at −100 °C. Wolfer [23] has succinctly summarized He bubble growth in metals as a function of homologous temperature, T*_h_*, and pressure. In the case of an isolated single bubble at temperatures below T*_h_* = 0.25, theory indicates that the bubble pressure is high enough for loop punching only at μ/5, where μ is the shear modulus of the material [24]. Below this pressure at T*_h_* = 0.25, bubble growth is not expected to occur. In Pd, μ = 42 GPa, making the pressure required for loop punching from an isolated single bubble 8.4 GPa. The NMR measurements discussed above [20] found bubble pressures of 6–11 GPa in the −173 °C to 77 °C range in 1 year old PdT_0.62_, very close to the threshold for loop punching.

When theory is applied to the case of a He bubble array, instead of an isolated bubble, the pressure required for loop punching increases to about 50% of the shear modulus [25]. Bubbles with radii less or equal to ~5*b*, where *b* is the Burgers vector of a prismatic dislocation loop, require a high enough He density to create bubble pressures sufficient for loop punching, independent of the bubble density. As bubbles grow by loop punching, the accumulation of loop debris in the regions between bubbles exerts an increasing and opposing force to the subsequent formation of loops, increasing the pressure required for loop punching dramatically. Thus, during the initial He accumulation period at cryogenic temperatures, one might expect slow growth by loop punching, but as the bubble density increases and the inter-bubble spacing decreases, the pressure required for loop punching likely becomes too high for additional growth to occur. This mechanism may have caused bubble growth to cease in this experiment, while bubble areal density continued to increase.

As the bubble pressure continues to increase beyond the window where dislocation loop punching is viable, inter-bubble fracturing may occur, possibly leading to blister formation and He release [23]. Blister formation has previously been observed after implanting bulk Pd with 300 keV He to 1 × 10^18^ ions/cm^2^ (70 at.% He at the peak) at −180 °C, a much higher He concentration that the total implanted dose in this work [1]. Theory suggests that, particularly below a bubble density of 3 × 10^18^ bubbles/cm^3^, equilibration of the chemical potentials for gas atoms in the bubble and in interstitial solution could result in He diffusing freely throughout the solid without being trapped inside a bubble, causing rapid gas release once a critical concentration is reached [25].

### 4.3. Hydrogen-helium Interactions

By performing in-situ He implantation in an ETEM, this work is uniquely suited to explore the interactions of H and He in a model fcc system. Point defects are introduced in the lattice during He implantation (see Figure 1b for the dpa profile). Hydrogen is known to interact with such defects in metals [26,27,28], which may have influenced the bubble nucleation and growth rates observed in this work. The binding energies of H to defects in Pd are lower than many other metals [29]. Experimentally determined binding energies of H to Pd defects are about: 0.15 eV (self-interstitial), 0.23 eV (vacancy), and 0.29 eV (He bubble) [27,28,29]. Hydrogen is expected to have been strongly bound to these defects in the present work, which was done at −100 °C (0.015 eV), and could increase the size of He clusters. In fcc metals, up to six H atoms can occupy a monovacancy [29], which could influence the trapping kinetics of He atoms to vacancies. Additionally, the presence of H in interstitial sites may influence the diffusion of interstitial He through the lattice, and therefore influence nucleation and growth kinetics. More simulation work is needed to verify potential effects of H on He bubble nucleation and growth.

## 5. Conclusions

Palladium metal was implanted with 10 keV ^4^He in-situ, at cryogenic temperature, in a H_2_ environment. No lattice expansion indicating β-phase hydride formation was observed. He bubbles 1.2 nm in diameter were observed to nucleate after 6 at.% He. Bubble size did not change with implantation time, but bubble density did increase. These initial experiments highlight the strength of the MIAMI-2 facility for in-situ TEM exploration of H_2_ interaction with He bubbles at various temperature extremes.

This preliminary work has highlighted the new combination of extreme environments (cryogenics, gas implantation, and reactive gas exposure) that can be explored during direct real-time observation within a TEM. Further work is needed to fully understand these initial observations. This future work would include comparison between in-situ He implantations in the presence and absence of H_2_ at the same temperature, for both ambient and low temperature, to deduce the effects of H_2_ and temperature on bubble formation, as well as development of methods to ensure hydride formation in the ETEM. This study points to a new multidimensional stressor approach to in-situ TEM experiments that permits greater understanding of the response to complex environments by materials.

## Supplementary Materials

The following are available online at https://www.mdpi.com/1996-1944/12/16/2618/s1, Figure S1: Under-focus image showing He bubbles during implantation, Figure S2: Over-focus image showing He bubbles during implantation.
