# Supplementary material for: Investigating Helium Bubble Nucleation and Growth through Simultaneous In-Situ Cryogenic, Ion Implantation, and Environmental Transmission Electron Microscopy
Source: Materials (Basel). 2019 Aug 16;12(16):2618. doi: 10.3390/ma12162618 (PMC6719068; doi:10.3390/ma12162618)
Supplement: Supplementary file 1 [file materials-12-02618-s001.pdf]

materials

Supplementary

Investigating Helium Bubble Nucleation and Growth through Simultaneous In-situ Cryogenic, Ion Implantation, and Environmental Transmission Electron Microscopy

Caitlin A. Taylor [1], Samuel Briggs [1,2], Graeme Greaves [3], Anthony Monterrosa [1], Emily Aradi [3], Joshua D. Sugar [4], David B. Robinson [4], Khalid Hattar [1,*] and Jonathan A. Hinks [3]

[1] Sandia National Laboratories, Albuquerque 87185, NM, USA
[2] Nuclear Science and Engineering, Oregon State University, Corvallis 97331, OR, USA
[3] School of Computing and Engineering, University of Huddersfield, Huddersfield HD1 3DH, UK
[4] Sandia National Laboratories, Livermore 94551, CA, USA
* Correspondence: khattar@sandia.gov

Received: 2 July 2019; Accepted: 13 August 2019; Published: date

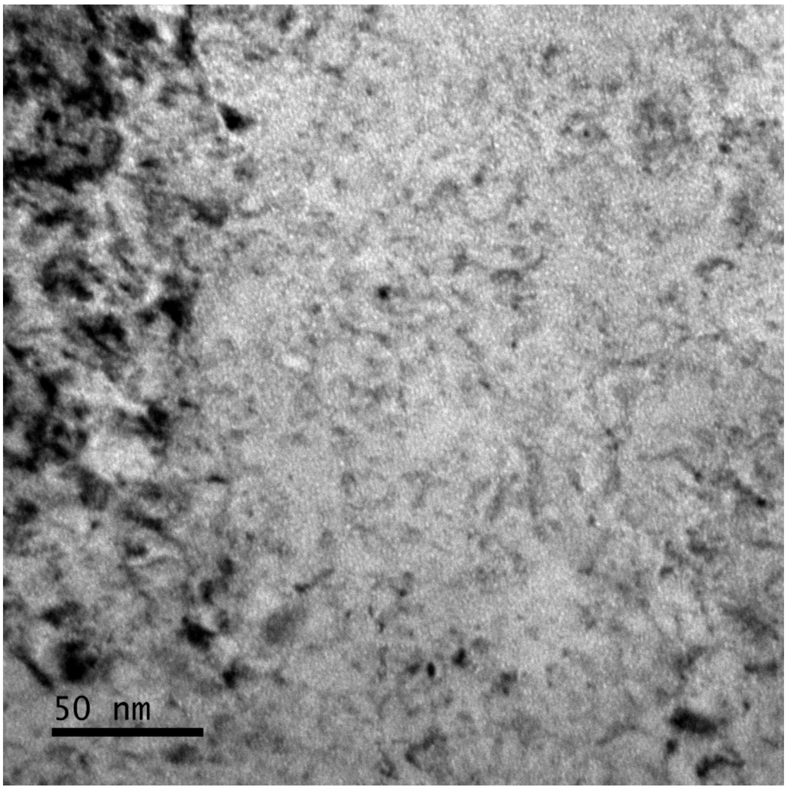

Figure S1. Under-focus image showing He bubbles during implantation.

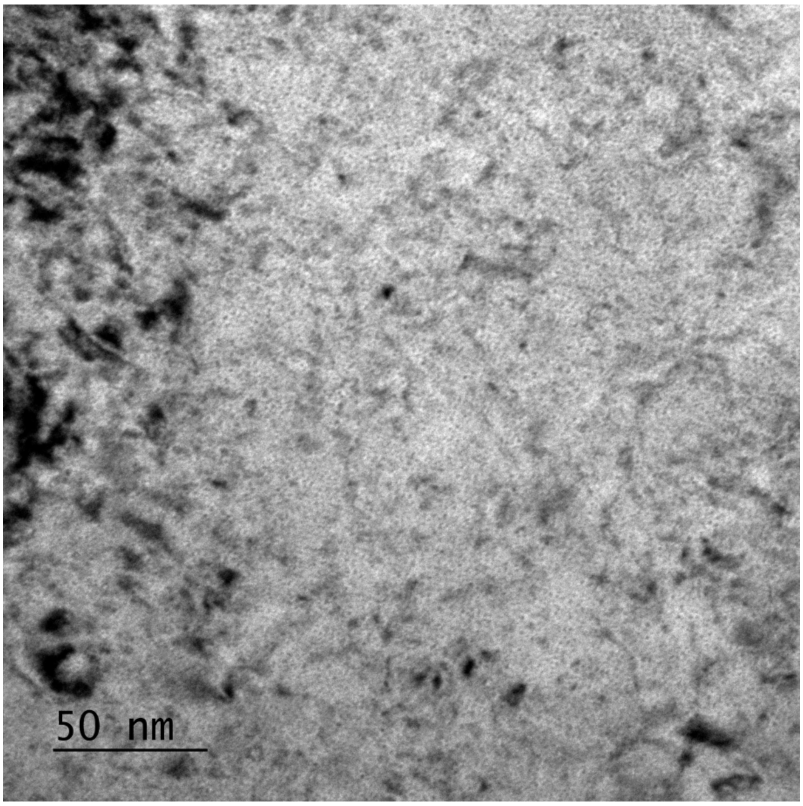

Figure S2. Over-focus image showing He bubbles during implantation.

© 2019 by the authors. Submitted for possible open access publication under the terms and conditions of the Creative Commons Attribution (CC BY) license (http://creativecommons.org/licenses/by/4.0/).
